# Development of a web-based resource for parents to support youth mental health: An exploratory sequential mixed methods approach

**DOI:** 10.1016/j.invent.2026.100961

**Published:** 2026-06-12

**Authors:** Sonia M. McCallum, Dominique Kazan, Alyssa R. Morse, Philip J. Batterham, Aliza Werner-Seidler, Michelle Torok, Sonja March, Cath Chapman, Tessa Reardon, Yael Perry, Bridianne O'Dea, Louise M. Farrer, Fiona Shand, Alison L. Calear

**Affiliations:** a Centre for Mental Health Research, College of Law Governance and Policy, The Australian National University, Canberra, Australia; b Black Dog Institute, University of New South Wales, Sydney, Australia; c Centre for Health Research & School of Psychology and Wellbeing, University of Southern Queensland, Brisbane, Australia; d The Matilda Centre for Research in Mental Health and Substance Use, The University of Sydney, Sydney, Australia; e Departments of Experimental Psychology and Psychiatry, University of Oxford, Oxford, United Kingdom; f The Kids Research Institute Australia, University of Western Australia, Perth, Australia; g Flinders University Institute for Mental Health and Wellbeing, Flinders University, Adelaide, Australia

**Keywords:** Child, Development, Digital intervention, Mental Health, Parent, Suicide

## Abstract

There is a lack of digital gatekeeper resources developed for parents to assist them in recognising and responding to mental health problems in their children and adolescents. We aimed to address this by exploring parents' mental health information needs, and to iteratively develop and design a resource incorporating their feedback. A three-phase iterative user-centred design process was conducted to (1) explore parents' information needs and preferences through a survey; (2) develop and revise resource content through interviews with parents; and (3) obtain feedback from parents regarding the resource prototype to improve it. An online survey of Australian parents (*n* = 631) confirmed that there was a demand for an online gatekeeper style resource that focused on anxiety, depression, self-harm and suicide in children and adolescents. Thematic analysis of 14 semi-structured interviews with parents applied three deductive themes and one inductively identified theme, which reinforced the resource content and led to the inclusion of additional topics (e.g. self-care) and the lived experience voice (e.g. stories and quotes). A prototype of the resource was developed and reviewed by nine parents and two State/Territory Education Department representatives. Overall, the resource was rated as suitable by the participants, although additional improvements were made to usability and content relevance. Given sampling limitations, it is important that the program is validated with a diverse sample of parents, to ensure it meets the needs of the broader population.

## Highlights

• Three phase user-centred design informed development of resource for parents.
• Survey of 631 parents confirmed strong demand for online gatekeeper tools.
• Parents want information on youth anxiety, depression, self-harm and suicide.
• Parent interviews shaped content, adding self-care and lived experience stories.
• Resource refined by parent participants reviewing prototype and providing feedback.

## 1 Introduction

Anxiety, depression, self-harm, and suicidal ideation are significant mental health challenges in children and adolescents (Lim et al., 2019; Polanczyk et al., 2015). Experiencing a mental health condition during childhood can have adverse health, legal, financial, and social outcomes across the lifespan (Copeland et al., 2015). Unfortunately, fewer than half of young people with a mental disorder receive professional support (Barican et al., 2022; Wang et al., 2023) and treatment is rarely adequate (Sawyer et al., 2019).

Younger children can have difficulties articulating their feelings and are reliant on parents or other caregivers (henceforth referred to as ‘parents’) to recognise potential mental health problems and respond appropriately. With age, there is increased self-recognition of a mental health problem, however adolescents are often reluctant to seek help, and are more likely to receive help if supported by their family (Rickwood et al., 2007). Given this, parents play a critical role in supporting their child's mental health but need the knowledge and skills to recognise potential mental health problems when they arise, identify when professional help is needed, and the skills and capacity to access timely care and support (Reardon et al., 2018).

Despite this important role, research suggests that many parents lack the knowledge and awareness needed to adequately support the mental health of their children and adolescents. A recent systematic review found that parents of children aged 4 to 12 generally had low levels of child mental health literacy, high levels of stigmatizing beliefs, and minimal awareness of available help (Maddox et al., 2025). Parents of adolescents have reported similar challenges, with 39.3% unsure where to seek help for their adolescent's mental health concerns (Lawrence et al., 2015). One way to address the lack of knowledge and skills in parents is through the development of a parent-specific gatekeeper program. Most commonly suicide prevention programs, gatekeeper training programs equip community members with knowledge of the signs and symptoms of mental health problems, how to intervene, and how to connect distressed people in their community to appropriate support (Burnette et al., 2015). Gatekeeper interventions have been shown to be effective at increasing mental health literacy, reducing stigma, and increasing self-efficacy in responding to individuals in distress (Burnette et al., 2015). Educating parents in this way to promote help seeking behaviour aligns with the Theory of Planned Behaviour (Ajzen, 1991). This theory posits that attitudes, subjective norms, and perceived behavioural control (self-efficacy), which are shaped by knowledge, influence intentions to act or change. When supported, these intentions can translate into behaviour change, which in this case is more timely help-seeking behaviour.

Engaging parents in interventions can be difficult, with online delivery often overcoming a number of barriers (Baker et al., 2017). Two recent studies evaluated the online delivery of mental health literacy interventions with parents (Havewala et al., 2023; McKay et al., 2022). The first evaluated the virtual delivery of Youth Mental Health First Aid training (Havewala et al., 2023), while the other assessed the online delivery of LivingWorks Start program (McKay et al., 2022). While both programs were effective in increasing self-efficacy, and help-seeking intentions at post-intervention, neither was explicitly developed for parents. (Buchanan and Holly, 2025; Havewala et al., 2023; Kusaka et al., 2022; McKay et al., 2022; Murphy et al., 2022; Torok et al., 2019) The aim of the current study therefore was to develop, using an iterative user-centred design, a gatekeeper resource specifically for parents that provided them with the knowledge and skills to recognise and respond to mental health problems in their children (aged 5–17) and access timely help and support.

## 2 Development

The initial framework proposed for the *Recognise, Respond and Support – A Parent's Guide to Youth Mental Health* resource was based on the typical structure of gatekeeper interventions, which includes providing information and strategies to recognise distress, respond to the individual, and refer them onto support (Burnette et al., 2015). Development of the current resource was iterative and involved a user-centred design. Incorporating the intended users within the design phase ensured the resource would effectively meet the needs of parents. The research employed a sequential dependent mixed methods design, with the multiphase approach guiding the development of the resource through the elaboration and refinement of the key gatekeeper concepts. A three-phase process was undertaken to explore parents' mental health information needs and preferences (Phase 1), incorporate the experiences of parents who had sought help for their child's mental health (Phase 2) and obtain feedback on the resource prototype (Phase 3) to improve the content and design of the online resource.

**Fig. 1:**
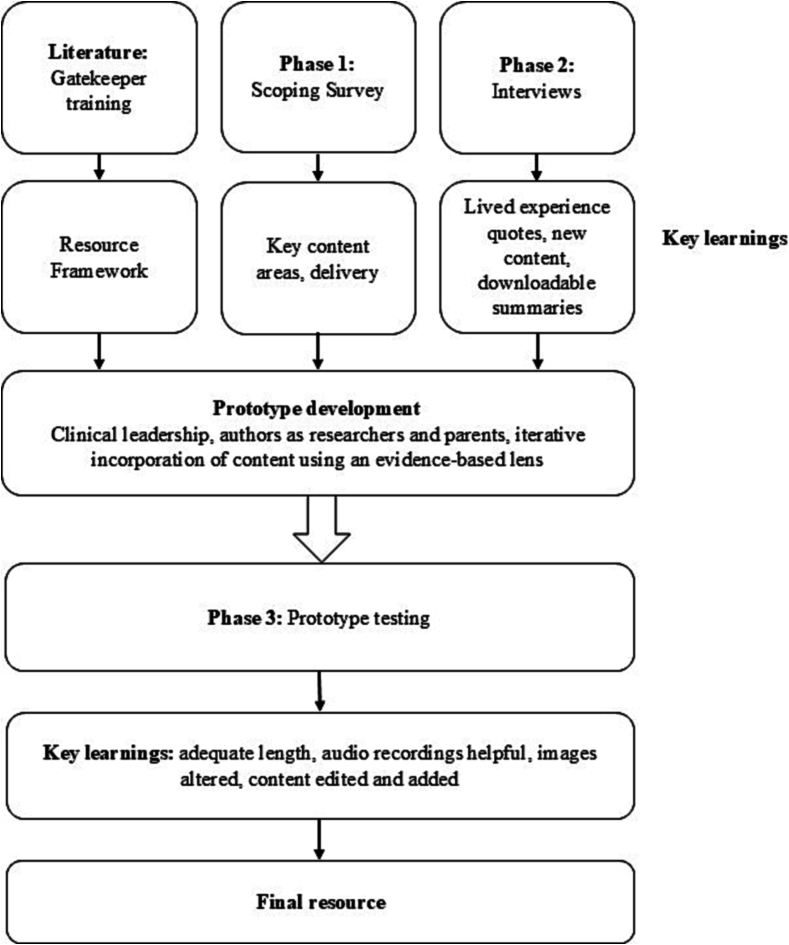
The development process for the Recognise, Respond and Support resource.

Phase 1 consisted of a community-based survey, which was used to scope the overall content and design of the resource. Initial resource content was then drafted by a clinical psychologist with expertise in child and family mental health, psychoeducation, and stigma reduction, and reviewed by team members with expertise in youth mental health, suicide prevention, and digital interventions. Members of the research team also drew on their own parenting experiences or those of their friends when developing resource content. Phase 2 involved interviews with parents who had sought help for a child with a mental health problem. This phase of the research helped refine existing resource content and identify new content areas, including lived experience examples. In Phase 3, a separate group of participants reviewed a prototype of the resource and provided feedback on its content and design, with particular emphasis on tone, readability, and overall flow. Each of these phases are described in detail below and depicted in Fig. 1. Ethical approval for this research was obtained from the Australian National University Human Research Ethics Committee (2021/440).

## 3 Phase 1 - scoping survey

Phase 1 employed a scoping survey to identify the mental health information needs of parents, and their preferences for receiving mental health information including accessibility, design features, and content. This phase was used to determine whether parents would access a gatekeeper-style program, and if so, what content and design features were desired.

### 3.1 Method

#### 3.1.1 Participants and procedure

A cross-sectional online survey was conducted with parents between 1st February and 27th March 2022. Participants were recruited using Australian parenting groups and paid advertising on Facebook. The advertisements invited parents to complete an anonymous 20-to-30-min survey to share their views on the features and content that would be helpful in a resource aimed at helping parents support the mental health needs of their children.

Participants were presented with a participant information sheet outlining their involvement in the study, which included the contact details of the researchers, and listed relevant mental health support services that could be accessed if required by the participant. Participants provided digital consent before completing a brief eligibility questionnaire confirming that they were aged 18 or older, lived in Australia, and currently had caring responsibilities for a 5–17–year old child. The survey landing page was accessed 1272 times with 798 participants providing consent, 768 (96%) meeting eligibility criteria, and 631 (82%) completing the survey. The median survey duration was 21 min. No incentives were offered for survey completion. Due to the exploratory aims of the survey, we did not conduct an a priori power analysis, therefore, the required sample size (*n* = 500) was based on recent social media recruitment experience, funding, and time constraints (Ma et al., 2023).

At the end of the survey, participants could express interest in participating in subsequent stages of the research project by clicking a link which took them to a separate survey. This collected their personal details (name, age, gender, email address), and details on their children (age, gender, whether the child had experienced a mental health problem, type of mental health problem experienced, and whether they sought help from a health professional). This information was collected separately to ensure scoping survey responses were anonymous. In total, 112 (17.7%) participants expressed interest and provided their details.

#### 3.1.2 Measures

A number of bespoke measures were created to assess parent preferences in terms of (1) disorders that could be covered in a resource (e.g., anxiety, autism spectrum disorders, depression), (2) age of target population (five age brackets covering 0–18-years), (3) mode of delivery (e.g., website, podcasts, booklets), and (4) resource use motivations (upskill before a problem arises, at the time a problem or concern is raised, support family or friends).

Participants' information needs were assessed by identifying the types of content they preferred (e.g., recognising signs and symptoms, initiating conversations, enhancing listening skills), preferred features (e.g., regular emails, videos, factsheets), and the relative importance of specific design elements (e.g., parental input, development by a reputable organization, interactivity).

Participants rated their self-efficacy in (1) identifying a mental health problem, (2) knowing how and where to seek help, (3) responding to a disclosure of suicidal ideation, and (4) learning from an online resource.

Finally, participant demographics were collected to enable assessment of participant diversity and previous experience with mental health problems in children and adolescents. A summary of participant characteristics is provided in Table 1.

**Table 1:** Participant characteristics.

Parameter	Frequency	%
Gender		
Woman	620	98.3%
Man	9	1.4%
Non-binary	2	0.3%
Language spoken at home		
English only	592	93.8%
English and another	38	6%
Another language only	1	0.2%
Location		
Metropolitan (capital city)	342	54.2%
Regional (other city/town)	224	35.5%
Rural or remote	64	10.1%
Currently in paid work		
Yes	530	84%
No	97	15.4%
Education level		
Less than year 12	12	1.9%
Year 12 or equivalent	24	3.8%
Certificate/diploma/associate	123	19.5%
Bachelor's degree	223	35.3%
Higher degree	249	39.5%
Marital status		
Single (never married)	24	3.8%
Married or de facto	523	82.9%
Widowed	4	0.6%
Divorced/separated	79	12.5%
Number of children		
1	199	31.5%
2	322	51%
3	86	13.6%
4	19	3%
5	5	0.8%
Child experienced a mental health problem		
Yes	501	79.8%
No	127	20.2%
	**Mean**	**SD**
Age *n* = 622 (range 29–66)	43.47	5.77

#### 3.1.3 Data analysis

Categorical data was analysed in SPSS v28 (IBM Corp, Chicago IL, USA) to generate descriptive statistics.

### 3.2 Results

#### 3.2.1 Participant characteristics

Table 1 reports the characteristics of the 631 participants included in the survey. The sample was predominantly female (98.3%), spoke only English at home (93.8%), in paid work (84%), and married or in a de facto relationship (82.9%). Most participants were tertiary educated, and just over half of the sample resided in a capital city. The average age of participants was 43.47 years. Participants had between one and five school-aged children, with half the sample having two school- aged children. Almost 80% of the sample reported that one of their children had experienced a mental health problem. Overall, the sample had 495 children aged 12 and under (63%) and 295 children aged 13 and over (37%).

#### 3.2.2 Mental health disorders and age range

From a list of 10 mental health problems, participants most frequently selected anxiety (*n* = 443, 70.9%), depression (*n* = 356, 57%), suicidal thoughts/behaviour (*n* = 186, 29.8%), and self-harm (*n* = 95, 15.2%) as their combined first and second priorities for parental learning (See Supplementary Table 1).

The majority of participants considered that parents were more likely to need to support the mental health of their children during middle childhood (9–12 years; *n* = 454, 71.9%), early adolescence (13–15 years; *n* = 528, 83.7%), and late adolescence (16–18 years; *n* = 391, 62%). Fewer participants considered a need in younger age groups: 22.7% (*n* = 143) for 0–4 years old and 44.5% (*n* = 281) for 5–8 years old.

#### 3.2.3 Delivery mode and resource use motivations

Table 2 presents participant preferences for mode of delivery of a resource for parents about child and adolescent mental health, with over half of participants preferring a website, mobile app, or online learning modules. Only two participants (0.3%) indicated that they were not interested in accessing this type of resource. Regarding motivations for use, the majority of participants (*n* = 575, 91.1%) reported they would access a resource on child and adolescent mental health when a concern or problem was identified by their child or another person. Over half of participants indicated that they would use the resource to gain knowledge and upskill before a problem or concern arose (*n* = 393, 62.3%), or to support a friend or family member to manage a mental health concern with their child (*n* = 358, 56.7%).

**Table 2:** Preference on accessing a resource.

	**N**	**%**
General website	448	71.0%
Mobile app	341	54.0%
Brief online learning modules	330	52.3%
Podcast	305	48.3%
A series of videos	238	37.7%
Booklet I can access online or have emailed	169	26.8%
Content by email	167	26.5%
Printed booklet	109	17.3%
Not interested at all	2	0.3%

#### 3.2.4 Content, features, and design aspects

Participants were presented with a list of 19 different content options that could be included within a resource (see Supplementary Table 2 for all options and responses). The five most frequently selected options were: how to recognise signs of mental health problems in children (*n* = 565, 89.5%), how to respond to mental health problems in children (*n* = 547, 86.7%), how to respond when your child tells you about their suicidal thoughts and other mental health difficulties (*n* = 518, 82.1%), information about where to seek help for your child's mental health problems (*n* = 497, 78.8%), and strategies to maintain open communication about mental health with your child (*n* = 480, 76.1%).

The types of features parents considered important in an online resource consisted of the use of scenarios (e.g., videos) demonstrating effective support strategies (*n* = 486, 77%), a frequently asked question section (*n* = 375, 59.4%), fact sheets about child and adolescent mental health that can be printed (*n* = 353, 55.9%), and short videos explaining content (*n* = 346, 54.8%) (for all options, see Supplementary Table 3).

Almost all participants responded that it was very/extremely important that the resource content be evidence-based (*n* = 618, 98.4%), developed with input from mental health professionals (*n* = 608, 96.8%), could be accessed and revisited at any time (*n* = 583, 93.1%), that it was easy to use (*n* = 576, 91.7%), and could be completed at their own pace (*n* = 556, 88.7%) (See Supplementary Table 4). Approximately two thirds of participants thought it was very/extremely important for the resource to include the experiences of other parents (*n* = 400, 63.6%) and be developed with input from other parents (*n* = 407, 64.9%). Fewer participants thought it was very/extremely important for the resource to encourage communication with other parents (*n* = 120, 19.1%), provide regular reminders to complete activities (*n* = 151, 24.1%), or be interactive (*n* = 155, 24.7%).

#### 3.2.5 Self-efficacy

Fewer than half of survey participants were confident that they could identify a mental health problem in their child and know what to do and where to seek help (Table 3). Only 36% were confident that they could appropriately respond to their child disclosing thoughts of suicide. The majority of participants (*n* = 525, 83.5%) reported being fairly or completely confident in their ability to learn from an online mental health resource.

**Table 3:** Self efficacy.

Self-efficacy - fairly confident or completely confident	N	%
Know what to do and where to seek help for a mental health problem in your child	288	45.6%
Identify a mental health problem in child	258	40.9%
Appropriately respond to your child disclosing thoughts of suicide	227	36.0%

### 3.3 Key learnings

The scoping survey identified that parents desired a digital resource, targeted at parents of both children and adolescents and which predominantly focused on anxiety, depression, self-harm and suicide. Many of the key components of gatekeeper training programs (e.g., symptom recognition, help-seeking sources) were endorsed as an area of need by parents. The information garnered from the scoping survey informed the overall design of the resource, including the delivery platform (online learning modules) and key content areas (recognition, response and referral).

## 4 Phase 2 – in-depth interviews

This phase employed semi-structured interviews with parents to gain an in-depth understanding of their experiences in supporting a child with a mental health problem. These interviews helped to identify what information would have been helpful for them in their journey from identifying a problem to accessing services, so it could be captured in the resource. Insights refined the resource and informed new content, including lived experience examples.

### 4.1 Method

#### 4.1.1 Participants and procedure

To ensure a range of experiences, participants were purposively sampled from those who completed the expression of interest survey at the end of the Phase 1 survey. To align with the resource, interview participant selection considered the child's (1) age, (2) gender, and (3) experience of anxiety, depression, self-harm, and/or suicidal thoughts or behaviours.

Thirty-nine participants were emailed an information and consent form with a reminder sent after two weeks. Semi-structured online Zoom interviews were scheduled with participants who returned a signed consent form. Of those contacted, 17 (42.5%) consented and 13 (76.5%) interviews were completed. Due to the lack of male participants, an additional male participant was recruited through targeted social media (Facebook) advertising, resulting in a total of 14 participants. Participants were mostly female (*n* = 13; 92.9%), reporting on their children who were female (*n* = 9; 64.3%), male (*n* = 4; 28.6%), or non-binary (*n* = 1; 7.1%). Parents reported that children experienced between one and six mental health problems (mea*n* = 2.6, *SD* = 1.4, mode = 1), although a formal diagnosis was not required. These included attention-deficit/hyperactivity disorder (ADHD; *n* = 3), anxiety (*n* = 12), autism (n = 1), depression (*n* = 8), disordered eating (n = 4), post-traumatic stress disorder (PTSD; n = 1), self-harm (n = 2), situational mutism (n = 1), and suicidal ideation (n = 1), with children aged between 3 and 15 years old when they first experienced a problem. This sample size was deemed appropriate based on the breadth of parent experiences shared.

All semi-structured interviews were conducted by the author, SMM, audio-recorded in Zoom, and ranged from 19 to 66 min (mean duration 42 min). A distress protocol ensured participant safety throughout the interview, with a clinical psychologist (DM) on call to talk with the participant if required and to support the identification of referral pathways if needed. During and at the conclusion of each interview, SMM recorded key details and emerging ideas. The interviews explored a parent's journey from finding out their child had a mental health problem, to seeking help and support. Questions explored parents' role in child mental health, how they became aware of any problems and their reactions, actions taken, barriers experienced in accessing professional help, information that was or would have been helpful, and the types of professional support perceived as was most helpful. Interviews concluded with a question about whether an online resource for parents would be helpful, along with the types of features to consider. After the interview, participants were emailed information on various telephone, internet and health professional resources they could access if required. Participants were also sent a $40AUD gift card for remuneration. Interviews were transcribed verbatim, with any identifying information removed.

#### 4.1.2 Data analysis

To identify and synthesise topics that would be helpful to consider in developing a resource for parents, a reflexive thematic analysis was conducted (Braun and Clarke, 2021). The analysis was deductive, with data mapped onto the three core components of a gatekeeper program – (1) recognising signs; (2) knowing how to respond; and (3) referral and help-seeking (Burnette et al., 2015). Simultaneously, data was inductively coded to identify additional overarching themes to consider in a resource for parents using the NVivo 12 software (QSR International). In addition, Microsoft Excel was used to record and categorise key concepts from each interview and support theme development. SMM maintained an audit trail within the Microsoft Excel document, recording her reflections on how the key concepts affected thoughts and considerations toward the development of the resource, and the outcome of each key concept on resource development (e.g. reinforced content, out of scope, considered, included, modified resource). While the entire interview was read and considered, only sections of text that directly informed the development of the resource were coded and notated. The analysis was led by SMM, a parent and researcher in child and adolescent mental health who regularly discussed and collaboratively reflected on developing codes, themes, and the impact on the resource development with ARM and ALC, both child and adolescent mental health researchers with ALC also a parent. The final analytical structure was developed through consensus, and illustrative quotes selected to support interpretation.

### 4.2 Results

Interviews were used to identify content that participants may have found helpful throughout their help-seeking journey, and their overall thoughts on the utility of a parent resource. Besides the three deductively applied themes, one additional overarching theme was inductively generated, *Self-care and support through the journey*. In addition, the title of the theme of *Knowing how to respond* did not consider the relationship between the parent and child, which affects the parent's response, therefore this theme was expanded to also consider the relationship with the child and changed to *Connecting and communicating with their child*. Within each theme, a total of thirteen sub-themes were identified as presented in Table 4, along with quotes to illustrate.

**Table 4:** Themes secerned from participant interviews.

Themes	Sub-themes	Example quotes
Recognising signs	What is normal vs early warning signs	“*one thing I really struggled with was figuring out what was normal adolescent behaviour and what was concerning. ... It's really… I struggle to find that, what's normal and what should you be concerned about, but in a way that doesn't make you panic*.” (Participant 6, female caregiver)
		“*I think I did miss the signs. I didn't, maybe wasn't on to it as I should have been. So, I think anything that can help families understand that, just what to look for.*” (Participant 14, female caregiver)
	When and how to respond	“*but at what point do you, if it's got a guidance about what point do you seek further assistance? I know that sounds very basic, like if you see these signs seek further assistance*,” (Participant 12, female caregiver)
		“*I didn't know what we were dealing with in the early years. Even a checklist, “does your child do this, this, this and this?”* (Participant 4, female caregiver)
	Disruption to family life as a trigger	“*disrupted our whole family and dynamic and way of life*” (Participant 5, female caregiver)
Connecting and communicating with their child	Connection and Communication	“… *you need to connect with what they're actually going through, rather than sort of put them in a box and give them a diagnosis*.” (Participant 6, female caregiver)
		“*So often it's through play or through having relaxing time together that you can have those conversations. It's not going to be as structured as you might like it to be.*” (Participant 2, female caregiver)
	Guidelines for a conversation	“*That's what I was thinking, you often get this very general advice. Like, ‘talk to your child.’ But actually, how do you frame it so you don't make it worse? I think we were worried about that too. Is there a script almost that you could use?*” (Participant 6, female caregiver)
		“*What to do when you notice these, how to have those conversations, those really open conversations in a way that the child doesn't feel confronted*.” (Participant 14, female caregiver)
	Open communication about mental health	“*So we've always normalised getting help*.” (Participant 5, female caregiver)
		“*I think we need to teach our children that it's OK to seek help if you're not feeling right, and that it's totally normal*.” (Participant 7, female caregiver)
Referral and help-seeking	Managing services and system (navigating the system)	“*So having a really good resource out there that actually understood the system would be fantastic. ... what is the pathway for different treatment options? I think that would have been really helpful, because I still find that at times very confusing.*” (Participant 2, female caregiver)
		“*… and I guess there needs to be a clear pathway. If this is happening, you see this person. We had to sort of find our own way*.” (Participant 5, female caregiver)
	Relationship with health providers	“*… with clinical psychologists and GPs, it's like anything, you need people who are specialised in areas, or you need to have that rapport. And you've got to keep moving around sometimes to get a rapport,*” (Participant 8, female caregiver)
		“*And as I experienced, you end up jumping around a little bit until you find one that you like or trust.*” (Participant 11, male caregiver)
	Waiting for help	“*Where to go that you're not going to be told, we can see you in six months*.” (Participant 14, female caregiver)
		“*So you're kind of left just waiting. … you're kind of left going, what do we do?*” (Participant 7, female caregiver)
	Medication	“*Now, at no point did a professional tell us how risky it is to change meds, and it was just never explained*.” (Participant 1, female caregiver)
		“*I was incredibly reluctant to put him on antidepressants because you have all these fears that your child is going to stop being themselves …*” (Participant 2, female caregiver)
Self-care and support through the parent journey	Self-care	“*… and I just didn't do anything for myself, and it's been a really hard road for us to recognise that self-care is so important.*” (Participant 2, female caregiver)
		“*That's what I decided quite a while ago, that you need to be the best you to be able to help. If you're not doing all that stuff, then you're not going to be any use*.” (Participant 7, female caregiver)
	Experiencing and addressing guilt and self-blame	“*There's something wrong with me because there is something wrong with my child.*” (Participant 8, female caregiver)
		“…*it's good to kind of hear other people's experiences, so you don't get caught up in that,* ‘*it's just us,’ kind of think*” (Participant 7, female caregiver)
	Support from family and friends	“*So maybe even if it has something on that page about if you and your partner have varying opinions on your child's mental health…, I think that would be a good idea as well*.” (Participant 13, female caregiver)
		“*When I spoke to other parents with their children who have anxiety, that can be the most validating thing.*” (Participant 4, female caregiver)

#### 4.2.1 Recognising signs

Many participants wanted more education on how to recognise a mental health problem in their children and adolescents and when to respond. Many mentioned the difficulty in distinguishing developmentally appropriate behaviour from early warning signs of mental health problems. Participants reported that having guidance would have helped them to respond and seek help earlier. Participants wanted tools to assess whether a problem warranted a response or professional help. Several participants suggested that a checklist or quiz about their child would have been helpful. In the interviews, participants reported that family dysfunction (e.g., “*a feeling of walking on eggshells*” (Participant 4, female caregiver)) or escalating problems at school were motivators to seeking professional help.

#### 4.2.2 Connecting and communicating with their child

Once participants were concerned or noticed a problem, the next step was communicating their concerns to their child. Within this theme, connecting with the child was an important step in helping the child to disclose how they were feeling. This was particularly relevant at the point of responding to a problem and seeking help. Some participants reported on their own realisations that finding times or places to connect with their child may help them to open up and communicate with them. While participants may have had knowledge themselves, they were concerned other parents may not know how to respond, particularly to certain behaviours such as self-harm, or have the language and knowledge of what to say without making it worse. Participants reflected that a script to guide conversations may have been helpful. Another approach reported by some participants was using their own mental health difficulties as a way to normalise their child's experiences, reduce stigma and foster a culture of help-seeking in their house.

#### 4.2.3 Referral and help-seeking

Accessing help and support was another challenge parents encountered in helping their child. All parents experienced challenges in navigating the mental health system. Experiences ranged from feeling at a loss and not knowing where to access help, lack of knowledge of what providers do or how to manage the various services their child was receiving, to what a crisis situation is. They expressed a desire, and believed other parents would benefit, from practical information such as available options, treatment approaches, how to initiate referrals, and guidance on booking extended general practitioner appointments for a mental health plan (which in Australia subsidises treatment). Alongside the challenges faced with the mental health system was the importance of the child's relationship with the health providers. Some participants needed to visit a number of specialists before their child developed a rapport with a mental health professional. Many parents experienced significant delays in accessing professional services and commented that it is important to know that there is a waiting period and what to do while waiting. Three participants talked about their experience with medication for their child's mental health problem and the stigma associated with their use.

#### 4.2.4 Self-care and support through the parent journey

Throughout the help-seeking journey, *self-care and support through the parent journey* was an important theme. Several participants reflected on the importance of self-care, particularly when they were deeply focused on their child's wellbeing and neglected their own needs. Often self-care was only considered when participants reached a point of desperation. Feelings of guilt and self-blame for their child's mental health problem were a common experience, and they wanted to know other parents had gone through something similar. Participants reflected on the need for support to help them understand their own experiences and know that they are not alone. Participants also reflected that support for the parent was facilitated and impeded by family and friends. Some felt unsupported by their partners and their parents and wanted access to information to share with them to enhance receptivity. Others mentioned they found support through friends with similar experiences even though it was hard initially, for example, one participant found support from a friend after she disclosed her family's crisis situation.

### 4.3 Key learnings

Participant interviews helped to further refine the focus and content of the resource. In particular, the interviews provided lived experience quotes that were added to the resource to normalise the experiences of parents and provide further insight into common thoughts and feelings. New content was also added to the resource on medication, to help parents better understand the role of medication in the treatment of mental disorders and to reduce the stigma often associated with its use in children. Information on the role of schools and seeking help and support in this environment was also added to the resource, as well as a section on self-care, its importance and potential pitfalls. Lastly, PDF documents of key content were created to enable users to save information for future reference, or to share with family and friends.

## 5 Integration of findings

On the basis of these findings, we developed and then refined an online mental health and suicide gatekeeper resource designed for parents to help them in supporting their child's mental health. The resource, titled *Recognise, Respond and Support – A Parent's Guide to Youth Mental Health,* provides evidence-based information and guidance, which was developed with input from mental health professionals, mental health researchers, and parents. It was designed for parents of children aged 5–17, the period when mental health concerns commonly emerge and when parents identified they most needed guidance and support. In addition to written content, short auditory content was created with young people, parents, and a psychologist to provide lived experience perspectives. Downloadable PDF summaries of key content were developed to enable information to be shared with family and friends. The focus of the resource was on the most prominent mental health conditions identified in the survey, broken into three components: *Recognise, Respond and Support*.

The *Recognise* section provides parents with an introduction to mental health using the ‘well to unwell’ mental health continuum, followed by specific information on anxiety, depression, self-harm, and suicide. Signs of anxiety and depression are provided in separate lists for children and adolescents, to ensure information is age specific. Guidance on when to seek help is also provided, noting the importance of symptom frequency and the effect of symptoms on child functioning.

The second component, *Respond*, provides parents with guidance on what to do if they are concerned about their child's mental health, including strategies to prepare for and initiate a conversation with the young person. This section directly addresses some of the concerns raised by parents in earlier phases of the research, including how to have a conversation about mental health and what to do if the young person is not interested in talking. The resource also outlines how to respond to self-harm and suicide with direct questions and a supportive, non-judgmental approach.

The third component *Support* commences with brief quotes from interview participants on how they felt when they learned their child was experiencing mental health difficulties. This is to normalise and reassure parents they are not alone in their feelings and experiences. Information on what professional support is available is provided, as well as guidance on seeking help and support from schools, self-care strategies, and promoting positive mental health conversations in the home.

## 6 Phase 3 – prototype testing

After refining the resource using Phase 1 and Phase 2 input, prototype testing was conducted. This Phase was designed to investigate the acceptability and usability of the resource within the target parent population, along with ensuring the content was appropriate, relevant and met their needs.

### 6.1 Method

#### 6.1.1 Participants and procedure

Participants were sourced from the expression of interest survey that followed the Phase 1 scoping survey. Participants who had not been contacted for the Phase 2 interviews were purposively sampled to ensure a range of experiences were captured, including participants with and without a child with a mental health problem with a range of ages, genders and mental health problems reported.

Twelve parents were emailed a participant information sheet and consent form, of which 7 (58.3%) returned a completed and signed consent form. Consenting participants were sent an email with the feedback document attached, a list of mental health support services that could be accessed if required and were informed to stop providing feedback if they began to feel distressed. Five (71.4%) participants returned feedback. To supplement participant numbers, four additional parents known to the researchers were also invited to participate in prototype testing. In addition, two Education Department representatives located in different jurisdictions provided prototype feedback to ensure intervention materials were relevant and adequately represented the support that could be provided in schools. Independent review of the resource and completion of the feedback form was estimated to take 4 to 5 h. Participants were given online access to the resource and had one month to provide written responses to open-ended questions about the resource. After receiving their feedback, they were remunerated with a $200AUD gift card. This sample size was deemed appropriate based on the breadth of comments received.

Participants were provided with a 15-page document with blank boxes and were asked to provide feedback in two steps. The first step involved reading the resource end-to-end and completing questions regarding their overall impression, duration to complete the resource, desired length, the tone used, reading level, and anything they would like to see added or removed. The second step was to go through the resource again and provide detailed feedback on each section, including content that could be added or removed, identification of unclear wording, appropriateness of reflection questions, content summaries, and use of interactive components and audios.

#### 6.1.2 Data analysis

Feedback was collated in Microsoft Excel and assessed according to the section headings on the feedback form. Collated data for each section of the resource was read by SMM multiple times to gain familiarity and to recognise similarities between- and within-participant comments and sections, which were noted and recorded. All comments were considered and enacted upon, unless they were not feasible (e.g., resource available in different languages), or were outside the scope of the current resource (e.g., information on eating disorders). Decisions on how to amend the content of the resource in response to the feedback was determined through collaborative discussion with the research team (SMM, ALC, and DM) and was based on alignment with other evidence and whether it addressed gaps in information that were considered important for parents. Decisions on how to revise the design features (e.g., images, colours, formatting) were addressed by SMM, ALC, and the Black Dog Institute design team.

### 6.2 Results

Participants took an average of 1.7 h to read the entire resource and commented that this timeframe seemed appropriate for this type of resource. Several participants recommended that users be informed to complete the resource over multiple sessions.

Most participants considered the tone of the resource to be balanced and the reading level appropriate, with content easy to understand, and avoiding excessive scientific or clinical language. Some sentences were highlighted as requiring revision to reduce length and improve grammar.

To enhance resource presentation and display, participants recommended using different images in some places to ensure alignment with content and reduce the corporate feel, or the inclusion of infographics to further convey content messaging. The consistent use of symbols and text boxes was encouraged to enhance resource structure and consistency, and additional use of colour to make it more inviting.

Throughout the resource, audio recordings from children, parents, and a psychologist were included to help personalise the resource and include a lived experience perspective. These recordings were highly regarded by almost all participants and thought to be relatable and remind users that they are not alone in their experiences. To enable access for hearing impaired users, one participant suggested the inclusion of transcripts. One participant considered it would be helpful to have audio earlier in the resource, while five suggested the inclusion of audio in the self-care section to provide an authentic example of a parent would be valuable.

Participants were asked whether a video of a parent and child discussing self-harm or suicide would be helpful. Many parents liked the idea of a video to help this section, however there were concerns expressed that it could appear staged depending on the interaction and if done by actors, limiting their impact (*n* = 3), while one participant thought it would make the resource too long.

Participants also identified additional content to be included in the resource, including: (i) information on the age at which a young person can consent to healthcare treatments without the parent being present; (ii) broader recommendations regarding how schools can help; (iii) links to additional services (with access times) and resources; (iv) explaining how to navigate the resource; and (v) information on planning ahead and navigating a potential crisis situation.

### 6.3 Key learnings

Additional content was created as guided by participants' comments. For example, additional detail was included around developing a family safety plan, which is a pre-determined set of directions to help guide actions and decision-making in a crisis. Audio recordings were kept, with one being moved to an earlier stage in the resource, while another was created for inclusion in the self-care section. An additional audio of how to navigate the resource was included in the introduction. Images were altered, and visual elements were added including an interactive graphic detailing what happens when a call is made to emergency services.

## 7 Discussion

This paper describes the iterative development of one of the first gatekeeper style resources developed specifically for parents: *Recognise, Respond and Support – A Parent's Guide to Youth Mental Health*. The views of over 600 parents were sought at three distinct phases to iteratively develop and refine the resource. Inclusion of personalized stories throughout the resource based on quotes from interviews and the auditory clips from the lived experiences of parents, children, and a psychologist, strengthen the resource and provide users with evidence that others have experienced similar problems. Resource development was overseen by researchers and clinicians.

Most participants expressed interest in an informational tool to help parents support their child's mental health. Over half of participants reported low confidence in recognising a mental health problem in their child or knowing how to respond effectively. Interview findings echoed this, with many participants describing difficulty distinguishing normal developmental behaviour from early signs of mental health problems, and uncertainty about how to respond or seek help. These findings align with previous research identifying limited parental knowledge as a key barrier to help-seeking for children (Reardon et al., 2017; Salloum et al., 2016).

Survey participants expressed a strong preference for a digital resource with content that was evidence-based, which may reflect concerns about misinformation on the internet and social media (Shoval et al., 2022). Parents were less interested in an app or an interactive program compared to a static web-based resource. This contrasts somewhat with previous research on preferences for engaging with mental health interventions in younger people (Kim and Stout, 2010) although the preference for web-based interventions was consistent with findings in general adult populations (Batterham and Calear, 2017). Furthermore, previous research suggests that adults seeking information generally prioritise straightforward content over interactive or personalized features, which are more relevant in therapeutic interventions (Batterham and Calear, 2017).

Feedback from phases 1 and 2 largely corroborated existing literature, nevertheless, this approach provided additional critical insights, for example by informing the age range to include, defining the mental health focus, and identifying important content and elements to include. It highlighted the importance of a dedicated self-care section, supporting evidence that parents of children at risk of mental health problems often neglect their own wellbeing while prioritizing their child's needs (Martin et al., 2025; Putkuri et al., 2024). Likewise, it guided the development of downloadable content for ongoing reference or sharing, which may be particularly important during the often lengthy period of waiting for a service to be available (Subotic-Kerry et al., 2025). Furthermore, incorporating lived experience audios and quotes was intended to foster empathy and validation, potentially shaping parents' attitudes and intentions (Liao et al., 2025). Finally, the prototype testing stage enabled further refinements of the resource.

Potential limitations of this research include the low level of male participation, despite multiple efforts to increase male recruitment. This limited engagement, common in parenting research, suggests the resource may not fully capture the informational needs of fathers, who are known to have lower levels of child mental health literacy and less positive help-seeking attitudes (Johnson et al., 2023; Maddox et al., 2025). Another limitation was the high proportion of participants who had a child with an existing mental health problem. The finding that parents already interested in mental health were more likely to participate suggests there was self-selection bias (Kazmierczak et al., 2023). While insights from these parents were valuable for understanding help-seeking challenges, further engagement with parents who have not yet encountered such difficulties would help ensure the resource is relevant to a broader audience. This is particularly important given that over half of surveyed participants indicated they would use such a resource proactively – to build knowledge and confidence before issues arise. Consequently, future work should clarify whether *Recognise, Respond and Support* is primarily intended for parents currently managing mental health concerns in their children, or for those seeking to prepare in advance, as each group may have distinct informational and support needs. Given the limited representativeness of the sample, it is important that the program is validated with a diverse sample of parents and caregivers to ensure it meets the needs of the wider population. While the topics informed by the scoping survey aligned closely with higher prevalence conditions (depression, anxiety, suicidal distress); lower-prevalence conditions may require separate resources with a stronger emphasis on treatment rather than prevention and early intervention.

In conclusion, this study demonstrates the value of this approach in developing a digital gatekeeper resource for parents. *Recognise, Respond and Support – A Parent's Guide to Youth Mental Health* addresses a significant gap in parental support by providing evidence-based guidance to enhance recognition of early mental health difficulties and appropriate response strategies. While designed for an Australian audience, much of the core content of the resource has international relevance and could be readily adapted to account for differences in health and education systems. A randomised controlled trial has recently been completed to assess the resources effectiveness and acceptability (Calear et al., 2024), representing the first rigorous evaluation of a mental health and suicide gatekeeper program designed specifically for parents. If effective, this resource could serve as a scalable, preventive tool to strengthen parental capacity and promote early intervention in child and adolescent mental health.

## Funding statement

This work was supported by an NHRMC Research Fellowship (1173146) awarded to ALC. TR is supported by a fellowship from The Prudence Trust and receives funding from the Oxford Health NIHR 10.13039/100014461Biomedical Research Centre at Oxford Health NHS Foundation Trust. YP is supported by a People Fellowship from the Stan Perron Charitable Foundation.

## Declaration of competing interest

The authors declare that they have no known competing financial interests or personal relationships that could have appeared to influence the work reported in this paper.

## References

[bb0005] Ajzen I. (1991). The theory of planned behavior. Organ. Behav. Hum. Decis. Process..

[bb0010] Baker S., Sanders M.R., Morawska A. (2017). Who uses online parenting support? A cross-sectional survey exploring australian parents’ Internet use for parenting. J. Child Fam. Stud..

[bb0015] Barican J.L., Yung D., Schwartz C., Zheng Y., Georgiades K., Waddell C. (2022). Prevalence of childhood mental disorders in high-income countries: a systematic review and meta-analysis to inform policymaking. Evid. Based Ment. Health.

[bb0020] Batterham P.J., Calear A.L. (2017). Preferences for Internet-based mental health interventions in an adult online sample: findings from an online community survey. JMIR Ment Health.

[bb0025] Braun V., Clarke V. (2021).

[bb0030] Buchanan M., Holly L.E. (2025). A systematic review of parents’ mental health literacy programs: examining program effectiveness and parent satisfaction. Child Psychiatry Hum. Dev..

[bb0035] Burnette C., Ramchand R., Ayer L. (2015). Gatekeeper training for suicide prevention: a theoretical model and review of the empirical literature. Rand Health Q.

[bb0040] Calear A.L., McCallum S.M., Kazan D., Torok M., Werner-Seidler A., O’Dea B., Morse A., Farrer L., Shand F., Batterham P.J. (2024). Randomised controlled trial of an online mental health and suicide gatekeeper resource for parents and caregivers: study protocol. BMJ Open.

[bb0045] Copeland W.E., Wolke D., Shanahan L., Costello E.J. (2015). Adult functional outcomes of common childhood psychiatric problems: a prospective, longitudinal study. JAMA Psychiatry.

[bb0050] Havewala M., Cixin W., Diksha B., Chronis-Tuscano A. (2023). Evaluation of the virtual youth Mental Health First Aid Training for Asian Americans during COVID-19. Evid.-Based Pract. Child Adolesc. Mental Health.

[bb0055] Johnson C.L., Gross M.A., Jorm A.F., Hart L.M. (2023). Mental health literacy for supporting children: a systematic review of teacher and parent/carer knowledge and recognition of mental health problems in childhood. Clin. Child. Fam. Psychol. Rev..

[bb0060] Kazmierczak I., Zajenkowska A., Rogoza R., Jonason P.K., Scigala D. (2023). Self-selection biases in psychological studies: personality and affective disorders are prevalent among participants. PLoS One.

[bb0065] Kim H., Stout P.A. (2010). The effects of interactivity on information processing and attitude change: implications for mental health stigma. Health Commun..

[bb0070] Kusaka S., Yamaguchi S., Foo J.C., Togo F., Sasaki T. (2022). Mental health literacy programs for parents of adolescents: a systematic review. Front. Psychol..

[bb0075] Lawrence D., Johnson S., Hafekost J., Boterhoven De Haan K., Sawyer M., Ainley J., Zubrick S.R. (2015).

[bb0080] Liao Y., Adams D.R., Owens C.M., Jensen J.D. (2025). empathy, validation, and branding: testing the theory of empathetic suffering. J. Broadcast. Electron. Media.

[bb0085] Lim K.S., Wong C.H., McIntyre R.S., Wang J., Zhang Z., Tran B.X., Tan W., Ho C.S., Ho R.C. (2019). Global lifetime and 12-month prevalence of suicidal behavior, deliberate self-harm and non-suicidal self-injury in children and adolescents between 1989 and 2018: a meta-analysis. Int. J. Environ. Res. Public Health.

[bb0090] Ma S.O.N., McCallum S.M., Pasalich D., Batterham P.J., Calear A.L. (2023). Understanding parental knowledge, attitudes and self-efficacy in professional help-seeking for child anxiety. J. Affect. Disord..

[bb0095] Maddox R., Berry K., Wan M.W. (2025). What do parents know and feel about mental health in young children? A mixed methods systematic review of global parental mental health literacy. Curr. Psychol..

[bb0100] Martin F., Dahmash D., Wicker S., Glover S., Duncan C., Anastassiou A., Docherty L., Halligan S. (2025). Systematic review with qualitative meta-synthesis of parents' experiences and needs in relation to having a child or young person with a mental health difficulty. BMJ Mental Health.

[bb0105] McKay S., Byrne S.J., Clarke A., Lamblin M., Veresova M., Robinson J. (2022). Parent education for responding to and supporting youth with suicidal thoughts (PERSYST): an evaluation of an online gatekeeper training program with Australian parents. Int. J. Environ. Res. Public Health.

[bb0110] Murphy D., Heary C., Hennessy M., O’Reilly M.D., Hennessy E. (2022). A systematic review of help-seeking interventions for parents of adolescents. J. Adolesc. Health.

[bb0115] Polanczyk G.V., Salum G.A., Sugaya L.S., Caye A., Rohde L.A. (2015). Annual research review: a meta-analysis of the worldwide prevalence of mental disorders in children and adolescents. J. Child Psychol. Psychiatry.

[bb0120] Putkuri P., Latva-Korpela I., Hakkinen M. (2024). Support needed by parents when a child’s mental well-being is threatened—a qualitative study of views of experts-by-experience. Scand. J. Caring Sci..

[bb0125] Reardon T., Harvey K., Baranowska M., O’Brien D., Smith L., Creswell C. (2017). What do parents perceive are the barriers and facilitators to accessing psychological treatment for mental health problems in children and adolescents? A systematic review of qualitative and quantitative studies. Eur. Child Adolesc. Psychiatry.

[bb0130] Reardon T., Harvey K., Young B., O’Brien D., Creswell C. (2018). Barriers and facilitators to parents seeking and accessing professional support for anxiety disorders in children: qualitative interview study. Eur. Child Adolesc. Psychiatry.

[bb0135] Rickwood D.J., Deane F.P., Wilson C.J. (2007). When and how do young people seek professional help for mental health problems?. Med. J. Aust..

[bb0140] Salloum A., Johnco C., Lewin A.B., McBride N.M., Storch E.A. (2016). Barriers to access and participation in community mental health treatment for anxious children. J. Affect. Disord..

[bb0145] Sawyer M.G., Reece C.E., Sawyer A.C., Hiscock H., Lawrence D. (2019). Adequacy of treatment for child and adolescent mental disorders in Australia: a national study. Aust. N. Z. J. Psychiatry.

[bb0150] Shoval G., Chiu J.C., Taylor J.H., Barzilay R. (2022). Making evidence-based knowledge accessible to parents to promote child mental health care. J. Am. Acad. Child Adolesc. Psychiatry.

[bb0155] Subotic-Kerry M., Borchard T., Parker B., Li S.H., Choi J., Long E.V., Batterham P.J., Whitton A., Gockiert A., Spencer L., O’Dea B. (2025). While they wait: a cross-sectional survey on wait times for mental health treatment for anxiety and depression for adolescents in Australia. BMJ Open.

[bb0160] Torok M., Calear A.L., Smart A., Nicolopoulos A., Wong Q. (2019). Preventing adolescent suicide: a systematic review of the effectiveness and change mechanisms of suicide prevention gatekeeping training programs for teachers and parents. J. Adolesc..

[bb0165] Wang S., Li Q., Lu J., Ran H., Che Y., Fang D., Liang X., Sun H., Chen L., Peng J., Shi Y., Xiao Y. (2023). Treatment rates for mental disorders among children and adolescents: a systematic review and meta-analysis. JAMA Netw. Open.

