# Supplementary material for: Development of a web-based resource for parents to support youth mental health: An exploratory sequential mixed methods approach
Source: Internet Interv. 2026 Jun 12;45:100961. doi: 10.1016/j.invent.2026.100961 (PMC13279478; doi:10.1016/j.invent.2026.100961)
Supplement: Supplementary file 1 — Supplementary tables [file mmc1.docx]

**Parent Resource Development Publication – Supplementary Material**

**Supplementary Table 1: Combined first and second preferences for mental health problem**

|  | **N** | **%** |
| --- | --- | --- |
| Anxiety | 443 | 70.9% |
| Depression | 356 | 57.0% |
| Suicidal thoughts/behaviour | 186 | 29.8% |
| Self-harm | 95 | 15.2% |
| Attention deficit hyperactivity disorder | 59 | 9.4% |
| Autism spectrum disorders | 38 | 6.1% |
| Substance use | 36 | 5.8% |
| Eating disorders | 23 | 3.7% |
| Conduct and Oppositional defiant disorder | 11 | 1.8% |
| Bipolar | 3 | 0.5% |

**Supplementary Table 2: Content parents would value**

|  | N | % |
| --- | --- | --- |
| How to recognise signs of mental health problems in children | 565 | 89.5% |
| How to respond to mental health problems in children | 547 | 86.7% |
| How to respond when your child tells you about their suicidal thoughts and other mental health difficulties | 518 | 82.1% |
| Information about where to seek help for your child's mental health problems | 497 | 78.8% |
| Strategies to maintain open communication about mental health with your child | 480 | 76.1% |
| Different types of treatments that are effective for improving mental health in young people | 467 | 74.0% |
| How to acknowledge and accept children's experiences and feelings | 461 | 73.1% |
| Information about when to seek help for your child's mental health problems | 454 | 71.9% |
| Information on how to help your child in a mental health emergency | 452 | 71.6% |
| How to initiate conversations about suicide and other mental health difficulties | 449 | 71.2% |
| Actions that may promote mental health in young people (e.g., sleep, physical activity, media use) | 434 | 68.8% |
| Managing the relationship with your child after they have told you about their suicidal thoughts and mental health difficulties | 412 | 65.3% |
| Exploring feelings and reactions on discovering your child is experiencing problems with their mental health | 403 | 63.9% |
| Exploring mental health problems in children | 389 | 61.6% |
| Ways to improve listening skills | 377 | 59.7% |
| Strategies to take care of your own mental health and wellbeing | 377 | 59.7% |
| Guidance on the type of information needed for an appointment with a General Practitioner, Psychologist, School, etc | 339 | 53.7% |
| Information about support groups for parents | 314 | 49.8% |
| How and what to share with other people in relation to your child's mental health problems | 290 | 46.0% |

**Supplementary Table 3: Features to include in an online resource**

|  | N | % |
| --- | --- | --- |
| Scenarios (e.g., videos) demonstrating effective support strategies | 486 | 77.0% |
| Frequently asked questions | 375 | 59.4% |
| Factsheets about child and adolescent mental health that I can print | 353 | 55.9% |
| Short videos explaining content | 346 | 54.8% |
| Interactive activities that help you explore the mental health needs of young people | 296 | 46.9% |
| Regular emails containing lesson summaries of the content | 191 | 30.3% |
| Interactive game that helps you learn about the mental health of young people | 132 | 20.9% |

**Supplementary Table 4: Important aspects of a resource for parents**

| **Very important/Extremely important** | N | % |
| --- | --- | --- |
| Information is based on good evidence | 618 | 98.4% |
| Resource is developed with input from mental health professionals | 608 | 96.8% |
| Being able to refer back to the resource at any time | 583 | 93.1% |
| Resource is easy to use | 576 | 91.7% |
| Resource can be completed at my own pace | 556 | 88.7% |
| The organisation designing the resource is reputable | 510 | 81.2% |
| Resource is developed with input from other parents | 407 | 64.9% |
| Resource is structured, guiding you through content | 405 | 64.6% |
| Resource includes experiences of other parents e.g., in case studies | 400 | 63.6% |
| Resource doesn't take up too much time | 367 | 58.3% |
| Resource is integrated with what my child is learning at school | 167 | 26.6% |
| Resource is interactive | 155 | 24.7% |
| Regular reminders are sent reminding you to complete activities | 151 | 24.1% |
| Resource encourages communication with other parents | 120 | 19.1% |
